# Iterative approach to model identification of biological networks

**DOI:** 10.1186/1471-2105-6-155

**Published:** 2005-06-20

**Authors:** Kapil G Gadkar, Rudiyanto Gunawan, Francis J Doyle

**Affiliations:** 1 Department of Chemical Engineering, University of California Santa Barbara, CA, USA

## Abstract

**Background:**

Recent advances in molecular biology techniques provide an opportunity for developing detailed mathematical models of biological processes. An iterative scheme is introduced for model identification using available system knowledge and experimental measurements.

**Results:**

The scheme includes a state regulator algorithm that provides estimates of all system unknowns (concentrations of the system components and the reaction rates of their inter-conversion). The full system information is used for estimation of the model parameters. An optimal experiment design using the parameter identifiability and D-optimality criteria is formulated to provide "rich" experimental data for maximizing the accuracy of the parameter estimates in subsequent iterations. The importance of model identifiability tests for optimal measurement selection is also considered. The iterative scheme is tested on a model for the caspase function in apoptosis where it is demonstrated that model accuracy improves with each iteration. Optimal experiment design was determined to be critical for model identification.

**Conclusion:**

The proposed algorithm has general application to modeling a wide range of cellular processes, which include gene regulation networks, signal transduction and metabolic networks.

## Background

A systems level understanding of highly complex biological systems requires an integration of experimental techniques and computational research [1]. Current molecular biology techniques can generate high-throughput quantitative data that support *in silico *research using mathematical models [2]. These models can be used to simulate and study the dynamic interactions among the components of cellular systems as well as the systems' responses to external perturbations and signals. Such tools offer enormous potential for understanding cellular functions at the organism level [3]. Mathematical models also serve as test beds for generating hypotheses and designing experiments to test them [4]. Furthermore, they provide bases for model-based product and process design applications. Useful insights and predictions have been obtained for several biological systems from computational modeling and analysis. A few examples include the metabolic network analysis of *Escherichia coli *growth on glucose and acetate [5], the MAP kinase signaling pathways [6] and caspase function in apoptosis [7], and bifurcation analysis of cell cycle in *Saccharomyces cerevesiae *[8].

An iterative process for model development and the testing of hypotheses has been proposed by many researchers in the field and was recently highlighted by Kitano [1]. A qualitative approach of this process is described in [2]. In addition, Rabitz and co-workers [9] have recently developed an iterative method for closed loop parameter identification in biochemical reaction networks. A global inversion algorithm was used to extract the parameter estimates that minimize the differences between model prediction and experimental data. Unfortunately, global search methods typically have high computational requirements, and thus, do not scale very well with the system size. In this work, a quantitative model identification is developed that effciently obtains parameter estimates and facilitates scalability to very large network sizes. A proposed strategy is described in Section 2, with an emphasis on the modeling element. The modeling strategy is decomposed into three main steps: (1) determining the connectivity of the biological network and the interactions of the sub-components, (2) formulating the kinetics of inter-conversion among the subcomponents, and (3) estimating the parameters in the rate equations. To the authors' knowledge, this work represents the first documented example of multiple iterations for model refinement using such a framework in systems biology.

The parameter estimation from experimental data remains the bottleneck in the model development [4]. Banga and coworkers [10] have compared several advanced deterministic and stochastic global optimization methods for parameter identification from available experimental data. It was observed that the traditional gradient-based optimization methods often failed to arrive at the global optimal solutions. Deterministic methods [11-13] can achieve global optimality for certain classes of problems, but there is no guarantee of convergence in finite time [14]. Stochastic strategies [14-16] can locate the parameter region containing the global solution with relatively better effciency, but global optimality is not guaranteed. Furthermore, both methods suffer from the large computational burden required, even for moderately sized problems. Moreover, the validity of model with the estimated parameters over the entire operating space remains to be determined.

Parameter identifiability tests should be performed prior to the estimation process to ensure that the parameter estimation problem is well-posed. Further, the identifiability tests assist in selection of optimal measurements. Several researchers [17-19] have developed methods to determine whether a parameter is "identifiable *a priori*", i.e., identifiable from a given experiment design using the available measurements. A similar concept known as "practical identifiability" is concerned with the achievable accuracy of the parameter estimates. The confidence interval for the model parameters are determined using the Fisher Information Matrix (FIM) [20,21]. Doyle and coworkers [22] have performed model identifiability studies for a gene regulatory network using gene expression data, in which the identifiability of the parameters was found to be strongly dependent on the driving function.

The final step in the iterative model development process is the design of "optimal experiments" that would provide rich experimental data for improving the parameter estimates. Experiments can also be designed for discrimination among competing model structures that translates to selection between multiple proposed mechanisms of cellular function. Asprey and Macchietto [23] have developed a strategy of optimal experiment design for model structural identifiability. The strategy was used to identify the kinetics of the reactions in the fermentation of *Saccharomyces cerevesiae*. Kremling and co-workers [24] propose several strategies for model discrimination to identify the correct reaction mechanism of a test metabolic network. Banga and coworkers [25] have formulated the optimal design problem, using a scalar function of the Fisher Information Matrix (FIM) as the performance index, for parameter estimation of nonlinear dynamic systems.

In this work, an iterative procedure for model identification is proposed and applied to the caspase-dependent apoptosis system. An optimal measurement set is determined using the Fisher Information Matrix (FIM). The parameter estimation from partial measurements is decoupled into two parts. First, the available measurements are used to estimate the profiles of all unmeasured concentrations and reaction rates using a State Regulator Problem (SRP) formulation. In the second part the concentration and rate estimates are used to determine the model parameter values. The SRP formulation in this work is an extension of the dynamic Flux Balance Analysis (dFBA) approach developed by Doyle and coworkers [26]. Finally, the model-based experiment design uses parameter identifiability and D-optimality criteria to obtain the optimal experimental procedure that would generate the most informative data for model refinement in the next iteration.

## Results

**Figure 1:**
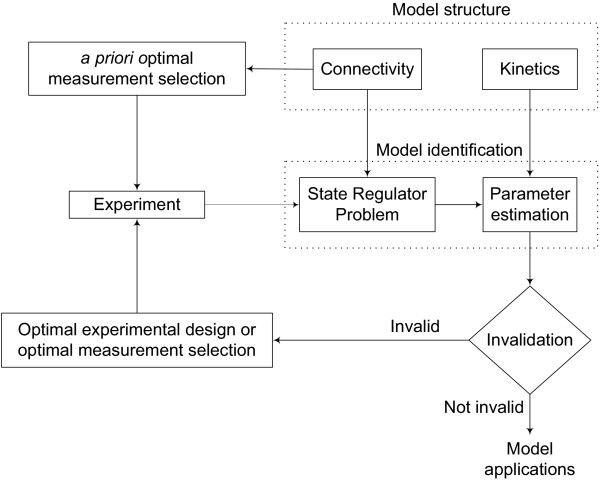
Iterative scheme for model identification.

The iterative scheme for model identification is shown in Figure 1. The optimal set of measurements is determined *a priori*. For an effcient model identification, a significant fraction of the unknown model parameters should be identifiable. In the case of poor identifiability, a higher number of measurements would be motivated. Also, the model complexity could be reduced to decrease the number of parameters; but this does not guarantee identifiability of the reduced number of parameters. Alternatively, a richer protocol (e.g. perturbation sequence [22]) might yield improved identifiability. In this work, selection of the *a priori *optimal measurement set is restricted to the "preliminary" experiment design that may be suboptimal.

The model connectivity and reaction mechanisms are developed from existing biological knowledge and are assumed to be known. The network connectivity, along with the partial measurements (optimal), is used in the State Regulator Problem (SRP) to obtain estimates of all system unknowns (unmeasured concentrations and reaction rates). Here it is important to note that the kinetics of the reaction rates are not used in the SRP algorithm. Next, the full estimates of the concentrations and the reaction rates are used for estimating the parameters in the kinetic model. This decouples the model identification into two parts such that the parameters involved in the kinetic equation of each reaction are independently determined as opposed to simultaneously estimating full model parameters from limited measurements. Next, the model invalidation test, which is a critical step in model development and the last "quality control" step before the desired application [27-29], is performed. Invalidity of the model could be determined by comparing model predictions with experimental data that is not used in the SRP algorithm. Further, model invalidity can occur if the model predictions conflict with documented biological knowledge of the system. In case of an invalid model, the model parameters are refined in subsequent iterations using the information obtained from the optimal experiment or by expanding the measurement set. The process of model identification is repeated in an iterative manner until an "acceptable" model is obtained.

### System

The mathematical model considered in this paper has the following structure:

 = *Ax *+ *Br *+ *C *    (1)

where

*r *= *f*(*x*, *p*),     (2)

*x *represents the protein/metabolite/gene concentrations, *r *the reaction rates, and *p *the model parameters. This is a very general nonlinear state space model in the variable *x*. The matrices *A *and *C *describe degradation and auto-generation respectively, whereas the matrix *B *represents the stoichiometry of the biological network. The kinetics among the proteins/metabolites/genes interactions show up in the reaction rates *r*. The aforementioned model is a continuous time invariant affine system from which a discrete version can be derived by standard techniques using a zero-order hold [30]. The resulting discrete model equation is represented as:



where



The discrete version of the model is used in the SRP estimation algorithm.

### Theory

#### Step 1: Determination of measurement set

The optimal measurement set consists of species whose concentration measurements would have maximum benefit for model identification, *e.g*, parameter identifiability and accuracy. In this work, the measurement set is determined such that the model parameters can be estimated accurately. The assessment of parameter identifiability in a model is crucial prior to parameter estimation from experimental data [31]. Identifiability is closely linked with parametric sensitivity analysis through the Fisher Information Matrix (FIM) [27]. The unidentifiable parameters are determined using the orthogonal procedure proposed by MacAuley and coworkers [19]. Here, a scaled sensitivity coeffcient matrix () shown below is computed:



where {*p*_1_, …, *p*_*k *_} are the model parameters, {*η*_1_, …, *η*_*m *_} are the response variables which include all possible measurable quantities, {*t*_1_, *t*_2_, …, *t*_*N*_} are the sampling times for the measurements, and  is the "initial" parameter value that is either the guess values of the parameter or the value obtained from literature. The orthogonal method is a geometric based approach where the number of identifiable parameters correlates with the rank of the orthogonalization of the scaled sensitivity matrix. The parameters corresponding to the columns of orthogonalized sensitivity matrix are deemed unindentifiable if the norms are smaller than a given tolerance. The details of the orthogonal method are not included here for the sake of brevity.

The next step is to obtain a measurement set that maximizes the expected accuracy in the identifiable parameters (practical identifiability). The Fisher Information Matrix (FIM) along with the Cramer-Rao theorem are used to determine a measurement set such that the estimated parameters have minimum variance. A detailed description of the procedure and its theoretical foundation can be found in [32]. Assuming that the measurement errors are additive and Gaussian, the FIM is given by [33]:

FIM = **J**^**T**^**WJ **    (5)

where **W **is the inverse of the measurement error covariance matrix and **J **denotes the sensitivity coefficient matrix for the measured response variables:



The quantity {*p*_1_, …, *p*_*r*_} denotes the identifiable parameter vector and {, …, } denotes the measured response variable vector.

The Cramer-Rao inequality establishes a lower bound on the variance of the identifiable parameters given by:

*σ*^2^(*p*_*i*_) ≥ (FIM^-1^)_*ii*_     (7)

The 95% confidence interval (CI) for a parameter is given by:

CI =  ± 1.96*σ*(*p*_*i*_)     (8)

In Equation (8) the lower bound of the variance is used. Symmetry of the confidence region about the nominal value is assumed. This results in the following definition of the percentage deviation from the nominal value:



The optimal measurement set is chosen such that the sum of the percentage error (*E*) for all the identifiable parameters is minimized. In this work, the optimal set is determined by a brute-force search over all combinations of measurement sets subject to restrictions that may be imposed by the system. Doyle and co-workers have developed effcient rational algorithms to determine the optimal measurement set with minimum computational burden [34]. The confidence intervals for non-identifiable parameters are infinitely large and hence are eliminated from the analysis. Identifiability for these parameters can be obtained only by a change in the experimental design or by the selection of an alternative model structure.

#### Step 2: State Estimation Algorithm

Generally, it is not possible to measure all time-varying components in a metabolic or signaling network. However, there are several techniques from systems engineering to estimate the behavior of unmeasured components given partial measurements of other system constituents. Bastin and Dochain have used an adaptive nonlinear observer for estimation of specific growth rate and biomass concentration [35]. Given accurate models, Extended Kalman Filters (EKF) have had success in several biological applications [36-38]. Artificial Neural Networks (ANN) have also found applications where dynamic models are not available [39-41].

In this work, an extension of Dynamic Flux Balance Analysis (dFBA) [26] is developed to estimate unmeasured concentration and reaction rate trajectories given partial measurement sets. The premise of this approach is straightforward: cellular processes have evolved regulatory structures that optimally use cellular resources. This premise translates into two postulates; (1) network flows are managed to minimize internal accumulation and (2) networks are managed to minimize the number of edges carrying flux at any given time. These two requirements are analogous to a classic problem in automatic control, namely, the State Regulator Problem (SRP). The SRP based estimator uses the measurement set selected from Step 1 to estimate unknown concentration and reaction rate trajectories via a constrained convex programming problem. The SRP estimator constrained by the key measurements captures the optimal cellular behavior of the system.

Estimates of the reaction rates at time step *k *and protein/gene/metabolite concentrations at time step *k *+ 1 are determined by the SRP and the discrete mass balance equations. The SRP must be solved at each sampling interval to obtain estimates of the unknown rates and concentrations. Consider the model (Equation 3); the discrete mass balance equations over a *p *step horizon are given by:



Where the matrices _*k*+1_, _*k*_, ^*x *^and ^*c *^are defined as



and the matrix ^*r *^is given by



The SRP estimator is a Quadratic Program (QP) with two cost terms, the cost of intermediate accumulation and the cost of operating a network reaction. The SRP penalizes intracellular metabolite or protein accumulation, but does not explicitly forbid it. Moreover, because reactions introduce an additional cost, the SRP only utilizes those reactions required to satisfy the mass balances and thermodynamic constraints. Formally, the estimation problem is given by:



subject to:

_*k* + 1 _≥ **0 **    (12)

*α*_*r *_(*k*) ≤ _*k *_≤ *β*_*r *_(*k*)    (13)



The SRP problem is subject to non-negativity constraints (Equation 12), flux-directionality constraints (Equation 13) and constraints imposed by the measurement set. Specifically, constraint of Equation 14 forces state estimates belonging to the measurement set to equal the corresponding measured value. The quantities  denote measurements that may have been corrupted with noise.  specifies the tolerance around the measurement within which the estimate is constrained to lie (incorporated to avoid numerical inconsistencies that may arise due to noisy measurements). The term Ξ^*X *^defines the measurement set. The matrix **W**_**x **_defines the cost of intermediate accumulation whereas the matrix **W_R _**represents the reaction cost:



For the caspase system considered in this work, the *w*_*x *_and *w*_*r *_are taken to be the order of magnitude of the inverse of the maximum value of the corresponding state or rate. This requires only approximate information regarding ranges of the protein concentrations and identification of the slow versus the fast reactions:





#### Step 3: Parameter estimation

The estimates of the concentration profiles and the reaction rates allow efficient determination of the parameter values by decoupling the full parameter estimation into multiple sets. Each set consists of parameters associated with one reaction rate. The parameters are obtained by minimizing the difference between the estimates of each reaction rate and that predicted by the kinetics *r *(*x, p*), which is a function of the concentrations. In case of the first iteration the minimization follows:



where *r*_*i *_are the individual reaction rates and *N*_*R *_is the total number of reactions. The kinetic parameters associated with a reaction rate equation are determined independently from those with other reactions, *i.e.*, the parameter estimation is decoupled with respect to each reaction.

For subsequent iterations, the Bayesian estimation formulation in [42] is used. In this formulation, in addition to the difference between the estimates of the reaction rates and the model predictions, the deviations of parameter values from those obtained after the previous iteration are minimized. The formulation can be represented as:



In the above equations,  is the estimate of the *i*^*th *^reaction rate and  is the estimates of the concentrations obtained from the SRP algorithm, *r*_*i*_(, *p*) is the predicted rate of the *i*^*th *^ reaction from the kinetics in Equation 2, *p *are the parameters associated with the *i*^*th *^ reaction rate, *p*_0 _are the parameter values obtained from the previous iteration, and *V*_*ε *_and *V*_*p *_are the variances of the estimates of the reaction rates and the prior parameter estimates. The parameter variances are determined using the Fisher Information Matrix (Equation 7). The variances of the non-identifiable parameters are infinite and penalty for deviations for these parameters are not considered in Equation 19. The variance for the estimates of the reaction rates can be determined from the expected noise in the measurements from which the estimates are obtained.

#### Step 4: Model invalidation tests

Given the iterative nature of this framework, a termination criterion must be established. Poolla et al. [29] have shown that for certain experimental data, it is not possible to confirm whether the model is really valid; however, one can conclude whether the model is not contradicted by the given data. Model (in)validation tests are usually based on the difference between the simulated and measured output and some statistics about these differences. Typical statistics for the model errors include maximum absolute value, mean value and variance [28]. In this work, model invalidity is tested by determining the model prediction errors using the estimated parameters. This error is calculated as:



To implement this test, experimental data that was not used in the SRP algorithm is required. The statistic used is the maximum and mean value of the errors for the measured states. When the prediction errors are below a certain desired value, the iterations are terminated.

#### Step 5: Model-based optimal experiment design

The optimal experiment design determines the optimal experiment to be performed for the next iteration such that there is maximum information content in the measurements. This would maximize the accuracy of the estimated parameters. The model-based optimal experiment design uses the Fisher Information Matrix as a measure of the amount of information contained in a given set of measurements about the model parameters [43]. The optimization searches through the space of experimental conditions or some parameterizations of the experimental protocol. For example, an optimal ligand input can be parameterized into a time series profile such that the optimization variables are the levels of ligand at different times (usually equally spaced in time). Naturally, the optimization will be restricted by the limitations in the experimental conditions and apparatus.

There exist several FIM-based optimality measures that quantify the overall informativeness of the measurements [32]. Among these, parameter identifiability and the D-optimality are the most widely used measures. For accurate model identification it is critical that maximum number of the parameters be estimated accurately. Thus, maximizing the number of identifiable parameters is the primary criterion proposed for determining the next experimental design. The orthogonal procedure proposed by McAuley and co-workers [19] is used to determine the number of identifiable parameters. There can be multiple experimental designs with the same maximum number of identifiable parameters. The selection among these is done so as to maximize the informativeness of measurement data. For this purpose, the D-optimality criteria is proposed. The use of D-optimality translates to minimizing the confidence interval of all the identifiable parameter estimates. The optimal experiment design criterion is shown as follows:



where **E **denote the feasible experimental conditions (defined by constraints in experiments), FIM is given in Equation 5 and *r *denotes the number of identifiable parameters. Thus, the identifiability is maximized in the sense that the hyper-dimensional confidence interval is minimized. For experimental designs with the maximum number of identifiable parameters, the one with the highest determinant of the Fisher Information Matrix is selected as the optimal design for the next experiment.

### A case study

**Figure 2:**
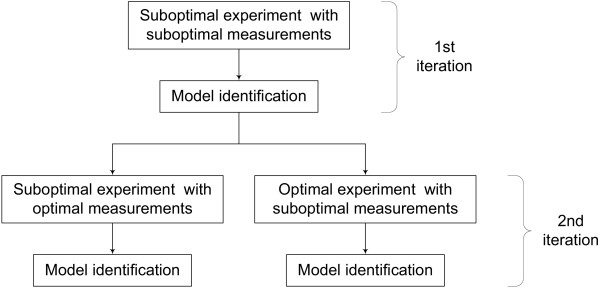
Sequence of simulations for model identification to test efficiency of optimal experiment design and optimal measurement selection.

The proposed iterative model identification is applied to the function of caspase-8 and caspase-9 in apoptosis. Caspase enzymes are at the core of the cell's suicide machinery. These enzymes are activated either by an external signal or by stress, and activated enzymes will then dismantle the cells. Varner and co-workers have developed a model for the caspase function in apoptosis [7]. The model describes the key elements of receptor-mediated and stress-induced caspase activations. The model consists of 19 states (enzymes) and 11 reactions with 27 parameters (11 rate constants and 16 saturation constants; see Appendix for additional details of the model). The *in silico *experiments in this study use the Varner model as the "actual" system. Measurements are assumed to be obtained from this "actual" system corrupted with up to 10% noise. The iteration starts using a "initial" parameter set, generated by perturbing the parameter values of the Varner model (considered as "exact") by 70–100%. The external and stress signals that activate the caspase system are considered as the manipulated variables in Step 5. Model refinement is performed either by determination of an optimal experiment or by optimal refinement of the measurement set. The performance of each of these criteria for model refinement requires to be tested. Therefore, in the first iteration, a "preliminary" suboptimal experiment with a suboptimal measurement set is considered. Moreover, in most cases of model identification, it is expected that preliminary experimental data is available. This data is usually obtained from a suboptimal experiment design and does not include the optimal set of measurements. The second iteration is performed in two ways; one by improving the measurement set and second by improving the experiment design. This tests the performance of both refinement criteria. The sequence of events is shown in Figure 2. It should be noted that it would be best to use optimal experiment design along with the optimal measurement set. However, it may not always be possible to do so due to feasibility issues specific to the particular system. Hence, this approach is not considered in this work. Model identification under less constrained conditions using a similar framework is included in [44].

**Figure 3:**
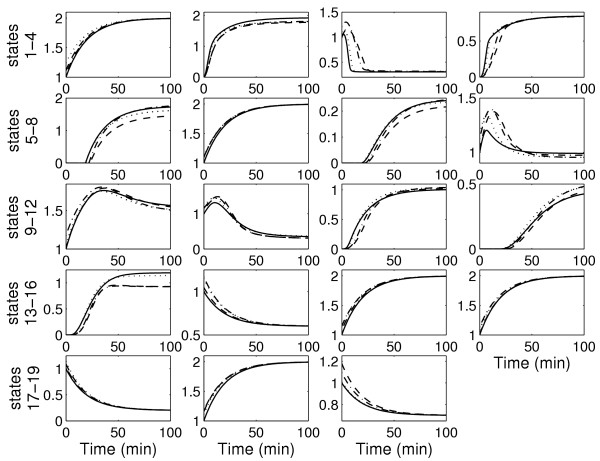
Prediction profiles of the 19 protein concentrations for the test experiment for the caspase system. Solid line: real system; dashed line: prediction with estimated parameters after first iteration (suboptimal experiment with suboptimal measurements); dash-dotted line: prediction with estimated parameters after second iteration (suboptimal experiment with optimal measurements); dotted line: prediction with estimated parameters after second iteration (optimal experiment with suboptimal measurements)

**Figure 4:**
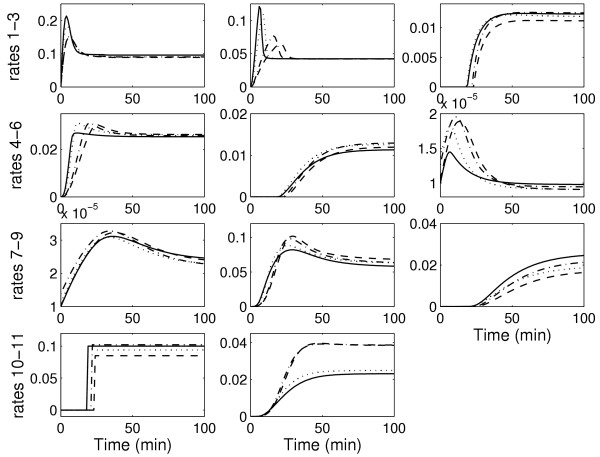
Prediction profiles of the 11 reaction rates for the test experiment for the caspase system. Solid line: real system; dashed line: prediction with estimated parameters after first iteration (suboptimal experiment with suboptimal measurements); dash-dotted line: prediction with estimated parameters after second iteration (suboptimal experiment with optimal measurements); dotted line: prediction with estimated parameters after second iteration (optimal experiment with suboptimal measurements)

After the first iteration, it is observed that there is a significant improvement in the predictions with the estimated parameters for both the protein concentrations and the reaction rates, as shown in Figures 3 and 4. The high errors with the "initial" parameters demonstrate that there is no bias in the results based on the starting guess values for the parameters and that there is indeed an improvement in prediction of both the protein concentrations and the reaction rates. However, the improvement is not suffcient as observed from the invalidation test (see Methods). This warrants a second iteration.

In general, the model predictions improve with the second iteration, as shown again in Figures 3 and 4. However, it is observed that the predictions are better for the case with the optimal experiment design, in spite of a suboptimal measurement set. This is due to the fact that model performance depends strongly on the accuracy of the estimated parameters. Using the suboptimal experiment, only 14 of the 27 parameters were identifiable. The optimal measurement set simply improved the confidence in these 14 parameters. On the other hand, the optimal experiment increased parameter identifiability to 18 parameters even with the suboptimal measurement set. These result indicates that performance of model identification is strongly linked with parameter identifiability.

## Discussion

In the proposed algorithm, the network topology and the mechanism of interactions in the pathway are assumed to be known. Several approaches have been proposed in literature to determine the network connectivity from experimental data [45-47]. In the case of unknown connectivity, these approaches should be used prior to the proposed model identification. The mismatch between the model and the actual network can appear in two different aspects of the algorithm. First, the SRP step can fail because there exist no feasible state and flux estimates that satisfy the measurement constraints. Such scenario arises mainly due to the network topology mismatch, and has low probability to occur due to the large degree of freedoms in a typical biological system. Alternatively, a well-designed model (in)validation step catches the mismatch between the model and the real system, *e.g.*, an independent measurement set contradicts the model prediction. Also, if multiple models are proposed for a particular biological process, model discrimination methods [24,48] can be used to identify the correct model structure. The effect of incorrect connectivity and/or mechanism would depend on the degree of mismatch and is case dependent.

The fact that cellular processes are carried out in an optimal manner lends tremendous promise to the success of this approach. In case the assumed optimality does not represent the *in vivo *behavior, the estimates from SRP may be inaccurate. However, the measurements (through the constraints in Equation 14) can attenuate this problem by restricting part of the estimates to match the observations. The parameter estimates may also be inaccurate if the real system deviates considerably from the assumed optimal behavior and this deviation is not captured by the measurements. The model identification framework has applicability to all systems that could be represented in the form shown in Equations 1 and 2. All biological processes are complex, interconnected networks. A feature common to these processes is that they have a fixed connectivity. The proposed algorithm for model development could be applied to metabolic networks, signaling processes and gene networks.

The computation burden for solving the SRP is minimal as it involves only a quadratic programming problem. The parameter estimation is also not computationally intensive due to the decoupling facilitated by SRP. A global optimization algorithm can also be used in the parameter estimation instead of the gradient search method to avoid convergence to local minima. However, the computation burden of parameter estimation will increase. To avoid local minima and high computation cost, the first few iterates (1 or 2) can utilize a global optimization method, while the remaining iterations can implement a gradient search algorithm. Due to the iterative nature of the approach, errors in parameter estimates can be tolerated as the corrections will be made in the next iteration with the optimal experiment. The optimal measurement selection is performed by a brute force search in this work. For very large systems the computation burden for this process grows exponentially. Computationally efficient algorithms for optimal measurement selection [34] can be used. Efficient measurement selection algorithms and the decoupling of parameter estimation for individual reaction rates into separate optimization problems result in good scalability properties of the proposed algorithm for large scale systems. One limitation of the approach is that it is dependent on the weights in Equation 15 for the minimization of the cellular resources. The choice of weights used in this work has provided accurate results but it requires information of the order of magnitude of the concentrations and rates of the system under study [44]. Efficient schemes for determining the weights for metabolic networks has been developed by Varner and co-workers [49].

## Conclusion

An iterative methodology for model identification from experimental data is developed in this paper. Identifiability tests are performed for an optimal measurement set selection for a given experimental design. The optimal measurements represent maximum information such that the model identification process is maximally benefitted. The model identification process is decoupled into two parts. In the first, the measurements are used to estimate all the unmeasured quantities of the system. This is achieved using the State Regulator Problem (SRP) formulation which is based on the assumption that the cell is an optimal strategist and uses its resources in an optimal manner. The SRP algorithm developed in this work has shown promising results. The average errors in the estimates for a significant fraction of the unmeasured responses is less than 10%. The accuracy of the estimates obtained by the SRP decreases with decrease in the information content due to suboptimal measurement set. In the second part, the full state and rate estimates are used to determine the model parameters. The decoupling relaxes considerable computation burden compared to estimating all model parameters simultaneously from the limited measurements. In the final step, a model-based experiment design determines the optimal experimental procedure that generates the most informative measurements for the next iteration. A strong dependence is observed between parameter identifiability and model performance. Thus, it is critical that the experiment design and measurement set be chosen such that maximum number of parameters are identifiable.

Tools developed for quantitative analysis of the dynamics of cellular pathways have tremendous potential in improving the predictive capabilities of biological systems especially in cases where experimental data is available but the kinetic parameters of the pathway reactions are unknown. The model developing tools are used for a host of applications and systems analysis.

The measurement selection algorithm presented in this work is freely available as part of a model analysis and development toolkit, BioSens [50].

## Methods

### Measurement set selection

The measurement selection analysis is performed using the "initial" parameter set for the "preliminary" experimental conditions shown in Table 1. A sampling time of 5 minute is assumed for a total simulation time of 100 minutes. Using the orthogonal procedure [19], the non-identifiable parameters are eliminated (13 of the 27 parameters). Perturbations of the non-identifiable parameters have no noticeable effect upon system dynamics for the given experimental protocol or have a strong correlation with the perturbation of one or more identifiable parameters. The non-identifiable parameters include the rate constants for auto-activation of the procaspases (parameters 5 and 6). The auto-activation is orders of magnitude lower compared to the activation by initiator. The small contribution of the auto-activation cannot be independently captured by the measurements and hence leads to non-identifiability. All other non-identifiable parameters are reaction saturation constants; the dynamics of which are not captured by the measurements at 5 minute sampling time for the given measurement noise. Equation 8 is used to estimate a bound on the confidence interval of the identifiable parameters and the deviation from the nominal value is calculated using Equation 9. A measurement set of 7 protein concentrations is assumed to be available. No measurements of the reaction rates are available. The choice of the measurement set was such that the maximum confidence was obtained for the identifiable parameters. The choice was made by a rigorous brute force search among all possible combinations. The optimal measurement set is shown in Table 2 and the confidence intervals are shown in Table 3. All the identifiable parameters have a confidence window with percentage error less than 30%. Further reduction in the percentage errors would require assuming more measurements in addition to the current 7 measurements, a faster sampling of the available measurements or a new experimental protocol.

**Table 1:** Experimental procedures used in model identification for the caspase system. Both receptor and stress signals are increased from zero to their maximum value in 30 minutes after which they are held constant (units same as in Varner model [7]).

	Maximum receptor signal	Maximum stress signal
Preliminary Experiment	0.24	0
Optimal Experiment	0.09	0.045
Test Experiment	0.15	0.030

**Table 2:** Measurement sets for the SRP estimator for the caspase system.

	States measured						
Optimal set	2	3	5	7	10	11	12
Suboptimal set	2	3	4	5	7	10	12

**Table 3:** Confidence intervals for the model parameters of the caspase system. Case 1: suboptimal experiment with optimal measurement set; Case 2: suboptimal experiment with suboptimal measurement set; Case 3: optimal experiment with suboptimal measurement set.

No.	Case 1		Case 2		Case 3	
	CI	% E	CI	% E	CI	% E
1	1.05 ± 0.25	23.31	1.05 ± 0.26	24.43	0.55 ± 0.04	07.18
2	1.65 ± 0.06	03.69	1.65 ± 0.06	03.50	0.95 ± 0.02	02.20
3	0.52 ± 0.01	02.45	0.52 ± 0.01	02.45	0.29 ± 0.04	15.53
4	1.69 ± 6e-3	00.34	1.69 ± 5e-3	00.32	1.69 ± 0.54	32.23
5	0.62 ± 0.01	01.81	0.62 ± 0.01	01.81	0.62 ± 0.14	22.38
6	NI	-	NI	-	NI	-
7	NI	-	NI	-	NI	-
8	0.87 ± 0.11	12.51	0.87 ± 0.11	12.48	1.33 ± 0.18	13.28
9	0.91 ± 0.25	27.87	0.91 ± 0.87	95.69	0.75 ± 0.44	57.85
10	0.15 ± 2e-3	01.49	0.15 ± 2e-3	01.49	0.09 ± 6e-4	00.70
11	0.23 ± 8e-3	03.39	0.23 ± 0.02	06.67	0.23 ± 0.04	17.69
12	2.12 ± 0.56	26.23	2.12 ± 0.59	27.59	17.8 ± 1.60	08.99
13	0.13 ± 2e-3	01.18	0.13 ± 2e-3	01.17	0.10 ± 8e-4	00.81
14	NI	-	NI	-	NI	-
15	NI	-	NI	-	14.7 ± 4.39	30.55
16	NI	-	NI	-	128 ± 30.8	24.03
17	NI	-	NI	-	NI	-
18	NI	-	NI	-	NI	-
19	NI	-	NI	-	127 ± 3.38	2.66
20	NI	-	NI	-	NI	-
21	(3.21 ± 0.06) × 1e3	01.76	(3.21 ± 3.14) × 1e3	97.51	(3.22 ± 1.16) × 1e3	36.05
22	NI	-	NI	-	NI	-
23	NI	-	NI	-	NI	-
24	NI	-	NI	-	NI	-
25	NI	-	NI	-	(3.09 ± 1.05)	33.95
26	0.79 ± 0.12	14.57	0.79 ± 0.12	14.52	1.04 ± 0.22	21.07
27	8.65 ± 0.31	03.60	NI	-	8.62 ± 3.62	42.04

### Model identification

#### 1st iteration

**Figure 5:**
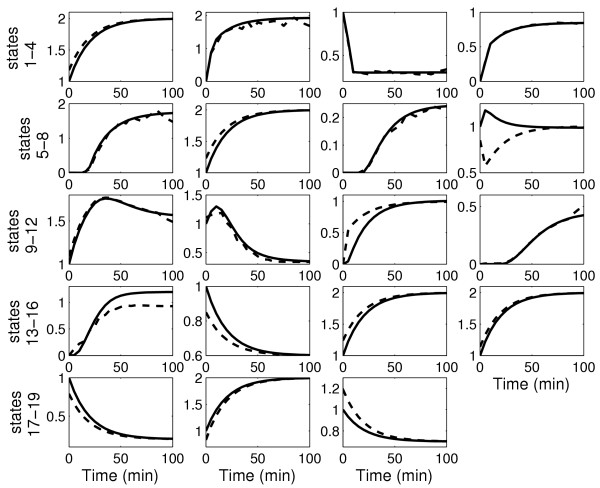
Profiles of the 19 protein concentrations for the caspase system. Solid line: actual system; dashed line: estimate by the SRP algorithm with the suboptimal experiment with suboptimal measurement set (first iteration)

**Figure 6:**
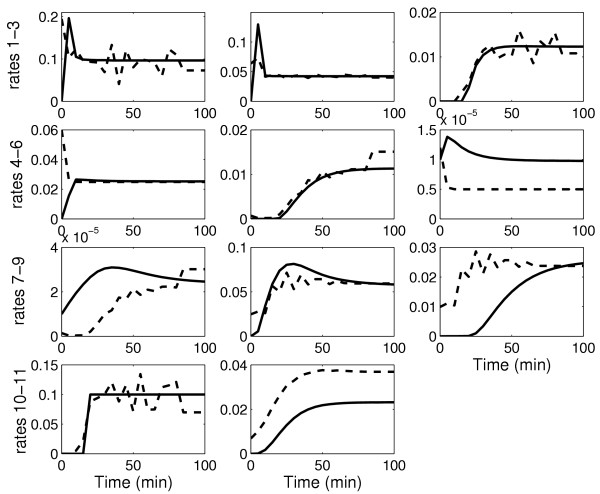
Profiles of the 11 reaction rates for the caspase system. Solid line: actual system; dashed line: estimate by the SRP algorithm with the suboptimal experiment with suboptimal measurement set (first iteration)

The "preliminary" experiment (Table 1) with a suboptimal measurement set (Table 2) is used for obtaining the estimates of the unknown concentration and reaction rate trajectories using the SRP algorithm. The sampling time is taken to be 5 minutes with a prediction horizon of 4. A higher prediction horizon showed no appreciable change in the estimates. The initial condition of the protein concentrations is assumed to be equal to the corresponding "actual" system corrupted by up to 25% relative error. Measurements are obtained from the "actual" system with up to 10% noise. The tolerance of the concentration measurements (14) is taken to be 5%. Figures 5 and 6 show estimated versus "actual" profiles for protein and reaction rate trajectories respectively.

The estimation error is determined by calculating the difference between the estimated and "actual" value for a rate/state at time *k *scaled by the maximum value over the entire simulation. Equation 22 shows the estimation error for concentration (the equation for reaction rates is identical). The scaling is done with respect to the maximum value in order to prevent misleading analysis at low concentrations or reaction rates:



The estimation errors (Equation 22) are given in Tables 4 and 5. Overall, it is observed that the estimates are fairly accurate and the system dynamics are captured. The average errors are less than 15% for all the state estimates and for most of the reaction rate estimates. Poor estimates, especially during the initial sampling times are mainly caused by the mismatch in the initial conditions. Further, the noise in the measurements results in fluctuations in some of the estimates. It should be noted that the estimation errors would not be available in real situations because the "actual" profiles are unknown. These are included here as a proof of concept.

**Table 4:** Estimation error for the protein concentrations in caspase system.

Protein (*x*_*i*_)	First iteration^*a*^		Second iteration^*b*^		Second iteration^*c*^	
	Max.	Avg.	Max.	Avg.	Max.	Avg.
1	08.93	01.91	08.93	01.91	08.93	01.91
2	12.49	04.74	10.54	03.55	12.04	04.32
3	03.64	01.38	04.02	01.31	03.27	01.40
4	02.16	00.82	02.38	00.78	01.94	00.83
5	14.63	03.95	12.46	03.35	13.87	03.62
6	11.54	02.47	11.54	02.47	11.54	02.47
7	09.04	02.58	07.48	02.07	08.47	02.27
8	51.10	10.65	12.65	04.40	51.63	11.24
9	05.11	01.46	05.11	01.58	05.09	01.49
10	10.22	04.18	08.45	02.35	09.02	03.65
11	48.30	09.41	07.79	02.74	50.75	10.49
12	18.56	02.76	17.67	02.59	18.19	02.69
13	22.27	14.66	22.10	15.40	13.13	03.45
14	15.00	03.21	15.00	03.21	15.00	03.21
15	12.04	02.58	12.04	02.58	12.04	02.58
16	07.02	01.50	07.02	01.50	07.02	01.50
17	21.00	04.50	21.00	04.50	21.00	04.50
18	08.42	01.80	08.42	01.80	08.42	01.80
19	19.00	04.07	19.00	04.07	19.00	04.07

^*a *^suboptimal experiment with suboptimal measurement set

^*b *^suboptimal experiment with optimal measurement set

^*c *^optimal experiment with suboptimal measurement set

**Table 5:** Estimation error for the reaction rates in caspase system.

Reaction rate (^*r*^*i*^)^	First iteration^*a*^		Second iteration^*b*^		Second iteration^*c*^	
	Max.	Avg.	Max.	Avg.	Max.	Avg.
1	98.97	15.98	103.3	15.91	67.05	14.14
2	49.09	05.68	48.76	05.68	45.11	06.54
3	30.86	11.60	30.46	11.64	29.49	11.93
4	225.9	14.86	38.71	18.08	221.1	16.25
5	34.32	10.44	33.49	09.79	33.87	10.30
6	61.73	38.69	66.30	41.42	65.93	38.27
7	79.40	37.65	80.00	38.12	81.26	37.20
8	35.03	10.47	36.67	10.75	36.03	11.34
9	112.1	38.78	109.9	37.32	112.5	36.55
10	34.50	17.92	36.56	17.92	29.43	10.44
11	76.59	62.44	72.72	60.33	29.76	10.03

^*a *^suboptimal experiment with suboptimal measurement set

^*b *^suboptimal experiment with optimal measurement set

^*c *^optimal experiment with suboptimal measurement set

**Figure 7:**
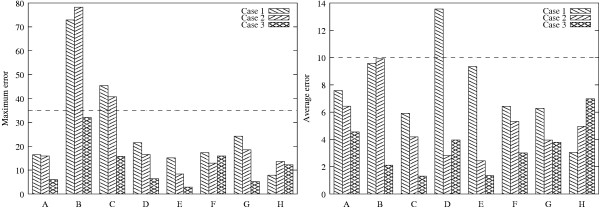
Maximum and average prediction errors for the measured protein concentrations. Case 1: first iteration (suboptimal experiment with suboptimal measurement); Case 2: second iteration (suboptimal experiment with optimal measurements); Case 3: second iteration (optimal experiment with suboptimal experiment). The measured protein concentrations are (A) FAS/FASL complex; (B) FADD; (C) FAS/FASL-FADD complex; (D) cytochrome c; (E) Apaf-1-cytochrome c complex; (F) executioner procaspase; (G) caspase-8; (H) caspase-9.

The estimates are then used to determine the model parameters by solving the optimization problem in Equation 18. The optimization to determine the parameters is a nonlinear program for the caspase system in which the model equations for the reaction rates are nonlinear with respect to the parameters. The nonlinear optimization is solved using the MATLAB routine *fmincon *which employs a gradient descent search method. As a starting point for the search, the "initial" parameter values are used. The optimization is solved for each reaction rate separately to obtain all the parameters in the model equations. Figures 3 and 4 compare the prediction profiles of the protein concentration and the reaction rates obtained with the estimated parameters with the "actual" profiles. These profiles are for an experiment condition ("test" conditions in Table 1) that is different from the one used for model identification. As a model invalidation test, the prediction errors are calculated using Equation 20. A threshold of 35% for the maximum error and 10% for the average error can be considered to be stringent. Figure 7 shows the result for the measured protein concentrations. It is observed that after the first iteration, the threshold is violated by the errors in the predictions of the FADD, FAS/FASL-FADD complex, and the cytochrome c. Tables 6 and 7 show the prediction errors for all the protein concentrations and reaction rates using the estimated parameters and also the "initial" parameters set. Again it is important to note that all these would not be available but are included here as a proof of concept.

**Table 6:** Error in the model predictions for the protein concentrations using the "initial" parameters, the estimated parameters after the first iteration, and the estimated parameters after second iteration for the "test" experiment of the caspase system.

(*x*_*i*_)	Initial parameters		First iteration^*a*^		Second iteration^*b*^		Second iteration^*c*^	
	Max.	Avg.	Max.	Avg.	Max.	Avg.	Max.	Avg.
1	11.21	02.26	05.66	01.14	02.54	00.51	12.04	02.43
2*	38.08	15.37	16.42	07.58	15.92	06.44	06.02	04.55
3*	90.59	16.37	72.90	09.57	78.27	09.91	32.07	02.11
4*	53.58	09.69	45.41	05.91	40.71	04.19	15.79	01.31
5*	75.88	27.09	21.47	13.56	16.54	02.81	06.40	03.96
6	01.74	00.35	04.82	00.97	04.76	00.96	05.52	01.11
7*	69.30	31.54	15.13	09.34	08.36	02.43	02.87	01.35
8	40.52	30.01	21.11	05.55	20.80	04.79	14.21	03.94
9	16.20	10.53	01.95	00.83	10.05	02.95	03.01	01.99
10*	56.81	38.13	17.30	06.43	12.93	05.32	15.92	03.01
11*	47.03	34.93	24.23	06.28	18.50	03.95	05.18	03.79
12*	66.80	36.63	07.88	03.04	13.61	04.96	12.22	06.98
13	53.52	39.26	22.14	14.01	22.25	13.75	04.66	02.79
14	11.13	02.24	04.75	00.96	17.06	03.44	15.18	03.06
15	11.69	02.36	07.36	01.48	03.80	00.77	07.90	01.59
16	05.84	01.18	00.72	00.15	06.79	01.37	04.65	00.94
17	10.47	02.11	09.19	01.85	03.77	00.76	14.38	02.90
18	10.61	02.14	07.92	01.60	08.75	01.77	05.66	01.14
19	13.13	02.65	17.94	03.62	09.46	01.91	01.10	00.22

^*a*^suboptimal experiment with suboptimal measurement set

^*b*^suboptimal experiment with optimal measurement set

^*c*^optimal experiment with suboptimal measurement set

*measured protein concentration for the invalidation test

**Table 7:** Error in the model predictions for the reaction rates using the "initial" parameters, the estimated parameters after the first iteration, and the estimated parameters after second iteration for the "test" experiment of the caspase system.

(*r*_*i*_)	Initial parameters		First iteration^*a*^		Second iteration^*b*^		Second iteration^*c*^	
	Max.	Avg.	Max.	Avg.	Max.	Avg.	Max.	Avg.
1	76.90	08.74	39.88	04.68	39.23	04.08	15.83	03.08
2	90.69	07.75	80.38	05.66	74.15	05.66	48.14	02.59
3	98.85	59.01	48.39	10.98	39.17	03.33	07.83	02.33
4	92.70	39.32	72.30	11.25	63.99	08.22	20.95	05.45
5	77.79	46.55	14.52	04.71	14.38	07.99	12.95	08.80
6	87.73	60.74	43.40	10.46	43.64	09.30	26.27	07.14
7	32.22	20.58	3.92	01.57	12.40	05.10	05.13	03.52
8	74.11	23.75	28.21	13.53	23.36	09.19	11.27	04.83
9	65.88	37.41	33.29	20.39	14.47	09.46	24.21	12.18
10	100.0	53.42	100.00	16.39	100.0	04.53	06.00	04.87
11	36.65	16.09	80.79	53.88	79.09	55.00	13.76	08.04

^*a*^suboptimal experiment with suboptimal measurement set

^*b*^suboptimal experiment with optimal measurement set

^*c*^optimal experiment with suboptimal measurement set

#### 2nd iteration

The second iteration is performed in two ways suggested in Figure 2. The first case involves using suboptimal experiment design with the optimal measurement set; whereas, the second case uses the optimal experiment with the suboptimal measurements. The model obtained after the first iteration is used to identify the optimal experiment that generate maximum information. For the selection of the optimal experiment design it is assumed that the measurement set remains unchanged. For the caspase system, the receptor and stress signals are parameterized such that both signals start at time 0 with constant rate injections reaching the maximum level in 30 minutes. The design variables are the final levels of the receptor and stress signals of the caspase activation. The search was constrained over a range of 0–0.4 for the receptor signal and 0–0.05 for the stress signal. A brute force search results in an optimal experiment (Table 1) with maximum information content for 18 identifiable parameters. The confidence interval for the identifiable parameters (Equation 8) and the deviations from the "nominal" parameter values (Equation 9) are shown in Table 3. Here it should be noted that the "nominal" parameters are the values obtained after the first iteration. The optimal levels for the signals suggest an optimal experiment with low receptor concentrations and high stress signal.

In each of the two cases, the model identification procedure is repeated in a similar manner as in the first iteration. The errors in the estimates of the protein concentrations and the reaction rates for both cases are shown in Tables 4 and 5 respectively. It is observed that the optimal measurement set improves the estimates of the states for which the information content increases. For example, the optimal measurement set includes caspase-8 (state 11), a state that is not included in the suboptimal measurements. The measurement of the caspase-8 improves the estimates of both the caspase-8 (state 11) and the procaspase-8 (state 8). The estimates are used to refine the parameter estimates using Equation 19.

Figure 7 shows the maximum and average prediction errors for the measured concentrations for the "test" experiment for the two cases in the second iteration. With the optimal measurements, although the overall errors are reduced, the threshold values are still violated. However, the predictions with parameters obtained from the optimal experiment reduce all the errors below the threshold. This support the termination of the iterative process with an acceptable model. The model prediction for all the concentrations and reaction rates for the "test" experiment are shown in Figures 3 and 4 and the prediction errors in Tables 6 and 7.

## Authors' contributions

KG performed the model identification work and drafted the manuscript. KG and RG performed the optimal experiment design and measurement selection work. FJD conceived of the study and participated in its design and co-ordination. All authors have read and approved the final manuscript.

## Appendix

**Figure 8:**
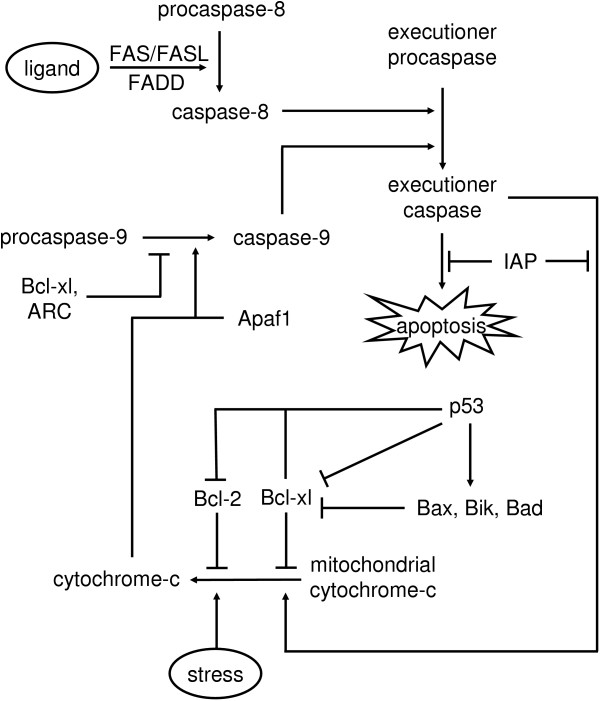
Caspase-dependent apoptosis mechanism. The model includes two triggers for the activation of cell suicide mechanism, extracellular death ligand and stress-related factor [7]. The cell death occurs when executioner caspase is activated by caspase-8 (ligand effector) or caspase-9 (stress-related effector).

The model of the caspase activated apoptosis proposed by Varner and co-workers [7] consists of 19 states (protein concentrations) and 11 reaction rates. Figure 8 gives the schematic of the apoptosis mechanism.

The model Equations can be represented as:



where *x*_*i *_denotes the ith protein concentration, *r*_*j *_denotes the jth reaction rate, Ω_*k *_denotes the rate of synthesis of the protein k and *μ *denotes the protein complex degradation rate. The reaction rates are as follows:



where



Tables 8 and 9 represent the nomenclature of the protein complexes and parameter values in the apoptosis model, respectively [7].

**Table 8:** Nomenclature in caspase-dependent apoptosis model.

No.	Protein complex name	No.	Protein complex name
1	total receptor ligands	11	caspase-8
2	clustered FAS/FASL complex	12	caspase-9
3	FADD	13	executioner caspase
4	FAS/FASL-FADD complex	14	decoy protein
5	cytochrome c	15	decoy protein
6	Apaf-1	16	decoy protein
7	Apaf-1-cytochrome c complex	17	activator protein
8	procaspase-8	18	Bcl-2
9	procaspase-9	19	Bcl-*x*_*L*_
10	executioner procaspase		

**Table 9:** Parameter values in caspase-dependent apoptosis model.

No.	Parameter	No.	Parameter	No.	Parameter	No.	Parameter
1	*k*_*l*_	8	*k*_83*a*_	15	*K*_*H*_	22	*K*_*K*_
2	*k*_*a*_	9	*k*_93*a*_	16	*K*_*I*_	23	*K*_*L*_
3	*k*_*h*_	10	*α*_*CE*_	17	*K*_*J*_	24	*K*_*N*_
4	*k*_8*za*1_	11	*k*_*u*_	18	*K*_*C*_	25	*K*_*O*_
5	*k*_9*za*1_	12	*K*_*S*_	19	*K*_*D*_	26	*K*_*P*_
6	*k*_8*za*2_	13	*K*_*A*_	20	*K*_*F*_	27	*K*_*R*_
7	*k*_9*za*2_	14	*K*_*B*_	21	*K*_*G*_		

## References

[B1] Kitano H (2002). Computational Systems Biology. Nature.

[B2] Ideker T, Thorsson V, Ranish JA, Christmas R, Buhler J, Eng JK, Bumgarner R, Goodlett DR, Aebersold R, Hood L (2001). Integrated Genomic and Proteomic Analyses of a Systematically Perturbed Network. Science.

[B3] Zhu H, Huang S, Dhar P (2003). The Next Step in Systems Biology: Simulating the Temporospatial Dynamics of Molecular Networks. Bioessays.

[B4] Cho KH, Shin SY, Kolch W, Wolkenhauer O (2003). Experimental Design in Systems Biology, Based on Parameter Sensitivity Analysis Using a Monte-Carlo Method: A Case Study for the TNF*α*-Mediated NF- *κ*B Signal Transduction Pathway. Simulation.

[B5] Edwards JS, Ibarra RU, Palsson BO (2001). *In silico *Predictions of *Escherichia coli *Metabolic Capabilities are Consistent with Experimental Data. Nat Biotechnol.

[B6] Schöberl B, Jonsson CE, Gilles ED, Müller G (2002). Computational Modeling of the Dynamics of the MAP Kinase Cascade Activated by Surface and Internalized EGF Receptors. Nat Biotechnol.

[B7] Fussenegger M, Bailey JE, Varner J (2000). A Mathematical Model of Caspase Function in Apoptosis. Nat Biotechnol.

[B8] Chen KC, Csikasz-Nagy A, Gyorffy B, Val J, Novak B, Tyson JJ (2000). Kinetic Analysis of a Molecular Model of the Budding Yeast Cell Cycle. Mol Bio Cell.

[B9] Feng XJ, Rabitz H (2004). Optimal identification of biochemical reaction networks. Biophys J.

[B10] Moles CG, Mendes P, Banga JR (2003). Parameter Estimation in Biochemical Pathways: A Comparison of Global Optimization Methods. Genome Res.

[B11] Esposito WR, Floudas CA (2000). Global Optimization for the Parameter Estimation of Differential-Algebraic Systems. Ind Eng Chem Res.

[B12] Grossmann IE (1996). Global Optimization in Engineering Design.

[B13] Papamichail I, Adjiman CS (2002). A Rigorous Global Optimization Algorithm for Problems with Ordinary Differential Equations. J Global Optim.

[B14] Guss C, Boender E, Romeijn HE Handbook of Global Optimization.

[B15] Ali MM, Storey C, Törn A (1997). Application of Stochastic Global Optimization Algorithms to Practical Problems. J Optim Theory Appl.

[B16] Törn A, Ali MM, Viitanen S (1999). Stochastic Global Optimzation: Problem Classes and Solution Techniques. J Global Optim.

[B17] Audoly S, Bellu G, D'Angio L, Saccomani MP, Cobelli C (2001). Global Identifiability of Nonlinear Models of Biological Systems. IEEE Trans Biomed Eng.

[B18] Jacquez JA, Perry T (1990). Parameter estimation: local identifiability of parameters. Amer J Physiol.

[B19] Yao KZ, Shaw BM, Kou B, McAuley KB, Bacon DW (2003). Modeling Ethylene/Butene Copolymerization with Multi-Site Catalysts: Parameter Estimability and Experimental Design. Polymer Reaction Eng.

[B20] Landaw EM, DiStefano III JJ (1984). Multiexponential, Multicompartmental, and Noncompartmental Modeling. II. Data Analysis and Statistical Considerations. Amer J Physiol.

[B21] Petersen B, Gernaey K, Vanrolleghem PA (2001). Practical Identifiability of Model Parameters by Combined Respirometric-Titrimetric Measurements. Water Science Tech.

[B22] Zak DE, Gonye GE, Schwaber J, Doyle III FJ (2003). Importance of Input Perturbations and Stochastic Gene Expression in the Reverse Engineering of Genetic Regulatory Networks: Insights from an Identifiability Analysis of an in Silico Network. Genome Res.

[B23] Asprey SP, Macchietto S (2000). Statistical Tools for Optimal Dynamic Model Building. Comput Chem Eng.

[B24] Kremling A, Fischer S, Gadkar K, Doyle III FJ, Sauter T, Bullinger E, Allgower F, Gilles ED (2004). A Benchmark for Methods in Reverse Engineering and Model Discrimination: Problem Formulation and Solutions. Genome Res.

[B25] Banga JR, Versyck KJ, Impe JFV (2002). Computation of Optimal Identification Experiments for Nonlinear Dynamic Process Models: A Stochastic Global Optimization Approach. Ind Eng Chem Res.

[B26] Mahadevan R, Edwards JS, Doyle III FJ (2002). Dynamic Flux Balance Analysis of Diauxic Growth in. E coli Biophys J.

[B27] Ljung L (1999). System Identification: Theory for the User.

[B28] Ljung L, Guo L (1997). The Role of Model Validation for Assessing the Size of the Unmodeled Dynamics. IEEE Trans Automat Contr.

[B29] Poolla K, Khargonekar P, Tikku A, Krause J, Nagpal K (1994). A Time-Domain Approach to Model Validation. IEEE Trans Automat Contr.

[B30] Brogan WL (1991). Modern Control Theory.

[B31] Vajda S, Rabitz H, Walter E, Lecourtier Y (1989). Qualitative and Quantitative Indentifiability Analysis of Non-Linear Chemical Kinetic Models. Chem Eng Commun.

[B32] Emery AF, Nenarokomov AV (1998). Optimal Experimental Design. Meas Sci Technol.

[B33] Beck JV, Arnold KJ (1977). Parameter Estimation in Engineering and Science.

[B34] Gadkar KG, Gunawan R, Doyle III FJ (2004). Heuristic Methods for Measurement Selection for Identification of Biological Systems. Int Conf Systems Biology, Heidelberg, Germany.

[B35] Bastin G, Dochain D (1990). On-Line Estimation and Adaptive Control of Bioreactors.

[B36] Albiol J, Robusté J, Casas C, Poch M (1993). Biomass Estimation in Plant Cell Cultures Using Extended Kalman Filter. Biotechnol Prog.

[B37] Gee DA, Ramirez WF (1996). On-Line State Estimation and Parameter Identification for Batch Fermentation. Biotechnol Prog.

[B38] Stephanopoulos G, San KY (1984). Studies on on-Line Biorector Identification. I. Theory. Biotechnol Bioeng.

[B39] Glassey J, Ignova M, Ward AC, Montague GA, Morris AJ (1997). Bioprocess Supervision: Neural Networks and Knowledge Based Systems. J Biotechnol.

[B40] Karim MN, Rivera SL (1992). Comparision of Feed-Forward and Recurrent Neural Networks for Bioprocess State Estimation. Comp Chem Eng.

[B41] Simutis R, Lübbert A (1997). Exploratory Analysis of Bioprocesses Using Artificial Neural Network-Based Methods. Biotechnol Prog.

[B42] Gunawan R, Jung MYL, Seebauer EG, Braatz RD (2003). Maximum a Posteriori Estimation of Transient Enhanced Diffusion Energetics. AIChE J.

[B43] Cover TM, Thomas JA (1991). Elements of Information Theory.

[B44] Gadkar KG, Varner J, Doyle III FJ (2005). Model Identification of Signal Transduction Networks from Data Using a State Regulator Problem. IEE Systems Biology.

[B45] Wagner A (2001). How to reconstruct a large genetic network from *n *gene perturbations in fewer than *n*^2 ^easy steps. Bioinformatics.

[B46] Gardner TS, Bernardo DD, Lorenz D, Collins JJ (2003). Inferring genetic networks and identifying compound model of action via expression profiling. Science.

[B47] Basso K, Margolin AA, Stolovitzky G, Klein U, Dalla-Favera R, Califano A (2005). Reverse engineering of regulatory networks in human B cells. Nat Genet.

[B48] Chen BH, Asprey SP (2003). On the Design of Optimally Informative Deynamic Experiments for Model Discrimination in Multiresponse Nonlinear Situations. Ind Eng Chem Res.

[B49] Frey AD, Kallio PT, Gadkar KG, Varner J (2005). Dynamic Flux Balance Analysis of VHb Expression in *Escherichia coli *MG1655. Biotechnol Bioeng.

[B50] Taylor S, Gunawan R, Doyle III FJ (2005). BioSens 2.0: Sensitivity Analysis Toolkit. http://www.chemengr.ucsb.edu/~ceweb/faculty/doyle/biosens/BioSens.htm.

